# Mid-infrared laser emission from Cr:ZnS channel waveguide fabricated by femtosecond laser helical writing

**DOI:** 10.1038/srep18365

**Published:** 2015-12-22

**Authors:** Ya-Pei Peng, Xiao Zou, Zhengyuan Bai, Yuxin Leng, Benxue Jiang, Xiongwei Jiang, Long Zhang

**Affiliations:** 1 Key Laboratory of Materials for High Power Laser, Shanghai Institute of Optics and Fine Mechanics, Chinese Academy of Sciences, Shanghai 201800, China; 2 State Key Laboratory of High Field Laser Physics, Shanghai Institute of Optics and Fine Mechanics, Chinese Academy of Sciences, Shanghai 201800, China; 3 University of Chinese Academy of Sciences, Beijing 100039, China

## Abstract

The operation of a mid-infrared laser at 2244 nm in a Cr:ZnS polycrystalline channel waveguide fabricated using direct femtosecond laser writing with a helical movement technique is demonstrated. A maximum power output of 78 mW and an optical-to-optical slope efficiency of 8.6% are achieved. The compact waveguide structure with 2 mm length was obtained through direct femtosecond laser writing, which was moved on a helical trajectory along the laser medium axis and parallel to the writing direction.

Mid-infrared laser sources (2–5 μm) have been extensively investigated due to a number of commercial, scientific, and military applications, such as remote chemical sensing, medical diagnostics, eye-safe laser radar, and environmental monitoring^1^,^2^. Transition metal (TM) ion-doped II–VI materials have been shown to possess high emission cross sections, ultra-broad tunability in the mid-IR range, negligible excited state absorption (ESA, transitions to the upper levels are spin forbidden), broad gain bandwidths, thermal conductivities approaching that of sapphire, and a capacity for room temperature operation^3^,^4^,^5^.

In 1996, L.D. DeLoach *et al.* first demonstrated the use of TM^2+^-doped wide bandgap II–VI crystals as laser gain materials in the mid-IR region^3^. The TM ion-doped II–VI crystals provided a low-energy optical phonon cut-off, decreasing the efficiency of non-radiative decay of impurities^6^. After these pioneering publications, a great deal of attention was focused on Cr:ZnSe and Cr:ZnS. Cr:ZnSe and Cr:ZnS exhibit high-power continuous wave (>10 W) outputs, wide tunability, and mode-locked outputs^5^,^7^,^8^,^9^,^10^,^11^,^12^,^13^. These studies indicated that the Cr:ZnS gain medium exhibits spectral characteristics similar to those of Cr:ZnSe and can therefore serve as a viable alternative to Cr:ZnSe. Moreover, ZnS offers a noticeably higher thermo-conductivity of 27 W/mK (compared to 19 W/mK for ZnSe), a higher thermal shock parameter of 7.1 W/m^1/2^ (5.3 W/m^1/2^ for ZnSe), an improved hardness of 160 Knoop (120 Knoop for ZnSe), and a lower thermo-optic coefficient (46 × 10^−6^ K^−1^) than that of ZnSe (70 × 10^−6^ K^−1^)^4^,^14^. The first continuous-wave (CW) Cr:ZnS laser was reported in 2002, with a diode-pumped version described by I. T. Sorokina *et al.*^15^,^16^. Recently, a 10 W CW output power was obtained using polycrystalline Cr:ZnS as an active element^17^. The Cr:ZnS material provided better thermo-optical and nonlinear-optical properties (d_eff_ = 8 pm/V for ZnS), which places Cr:ZnS as a superior host material for high-power scaling femtosecond applications in the mid-IR range.

However, thermal lensing due to the presence of high thermo-optic coefficients occurs in the gain media under high pump irradiances, causing optical damage and even cavity instability that leads to a decrease in laser power. This thermal issue can be addressed through appropriate choices of heat removal techniques, pump wavelength, crystal geometry, and master oscillator power amplifier (MOPA) configurations^18^. Nonetheless, thermal effects have limited further power scaling. One solution is to utilize the waveguide geometry in order to greatly reduce the difficulties caused by thermal lensing in the gain medium.

Femtosecond-laser microfabrication has arisen as one of the most efficient techniques for direct 3D micro-machining of transparent optical materials^19^,^20^. The pulse energy is absorbed through nonlinear process lead to micro-explosions take place at the focal point of the laser pulse. It is inducing volume expansion in the optical breakdown tracks and residual stress in the surrounding regions in a very short time that does not allow the fast heat-transfer process^21^. This energy transfer can lead to a localized change in the refractive index, which can be exploited for the fabrication of waveguide structures. Because of this feature, focused femtosecond (fs) laser pulses produce localized modifications at the micrometer scale in the focal volume inside the material, in which either permanent or very stable changes in refractive index may be obtained^22^. The first report on fs laser-written waveguides in a family of glasses was presented by Davis *et al.* in 1996^23^. Subsequently, numerous reports have focused on waveguide fabrication in various transparent materials^24^,^25^,^26^,^27^,^28^.

The fs laser-inscribed waveguides can be divided into four categories: single line, double-line filament, cladding, and ridge waveguides^29^. Waveguide lasers exhibit interesting features, such as compactness, improved output performances, environmental robustness, and a low emission threshold, which is an attractive solution to the thermal lensing problem^30^. Channel waveguides have been developed in Nd:YAG ceramics and crystals, Nd:YVO_4_, Yb:YAG, Cr:ZnSe, and Cr:ZnS^1^,^18^,^31^,^32^,^33^,^34^,^35^, which provide the potential for lower emission thresholds and maintain high gains over longer propagation distances. Efficient laser emission was reported using these waveguides when pumped with tunable Ti-sapphire lasers or diode lasers^27^,^36^,^37^.

In this paper, we report the use of a mid-infrared ~2.3 μm laser in a Cr:ZnS channel waveguide that was prepared through direct fs laser writing with a helical movement technique. This system exhibits the potential for creating compact and stable environmentally sources.

## High-optical-quality Cr:ZnS preparation

**Figure 1:**
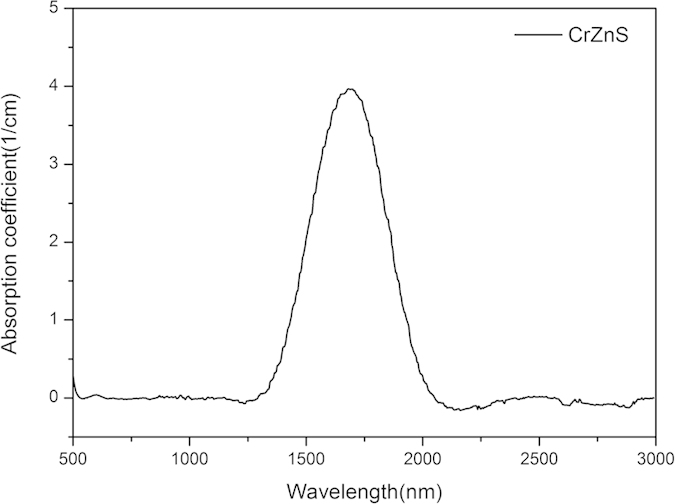
The absorption coefficient spectrum of Cr:ZnS.

High-optical-quality Cr:ZnS polycrystals were prepared using a typical post-growth thermal diffusion procedure. The chemical vapor deposition (CVD) grown ZnS polycrystals were deposited 0.5–1 μm-thick chromium metallic film on the sample surfaces. These were sealed to form square-shaped tubes of quartz under high vacuum conditions (<10^−5^ Torr) with high purity Ar (99.999%). These tubes were then placed in an 880 °C furnace and diffused for 5 days in order to obtain a uniform Cr distribution. The absorption coefficient spectrum of the prepared Cr:ZnS substrate is shown in Fig. 1, which implies that the polycrystalline Cr:ZnS substrate contains 4 × 10^19^ cm^−3^ Cr^2+^ ions, as per the relation c = α/σ_abs_. All the samples were polished to a size of 5 × 5 × 2 mm^3^ before further examination.

## Cr:ZnS channel waveguide fabrication

**Figure 2:**
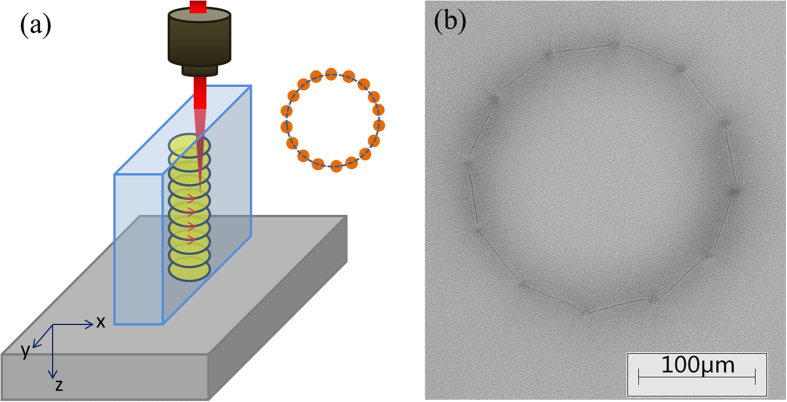
(**a**) Technique for direct fs laser writing using helical movement parallel to the laser medium axis. (**b**) Circular cross section of the channel waveguide design.

We used a scheme in which the Cr:ZnS laser polycrystal was moved along a helical trajectory during the writing process^38^. Compared with the classical translation method of waveguide inscription, helical movement of the laser medium during inscription can achieve a lower propagation loss in the waveguide structure^38^. The writing direction was aligned parallel to the laser emission direction, as shown in Fig. 2(a). The stage moved at a speed of approximately 1 mm/s in the horizontal direction, with a minor lift of 20–40 μm for each cycle in the vertical direction. For 3D processing, the movement of the stage was controlled by a computer and moved along with a mechanical shutter. The laser medium was moved circularly in the xy plane and translation was performed along the z direction, which provided a circular wall for the waveguide. We used a mode-locked Ti:sapphire laser with a wavelength of 800 nm and a pulse duration of approximately 130 fs (with a 1 kHz repetition rate and a 1 mJ maximum pulse energy). The fs laser pulse energy was controlled by a combination of a half-wave plate, a polarizer, and calibrated neutral density filters. The laser pulses with an average power of 120 mW were focused using a 20× objective lens with a numerical aperture of 0.4 and working distance of 15.3 mm into the polycrystalline Cr:ZnS substrate. The laser produced an ablation waveguide structure with a length of approximately 2 mm. Structures with diameters of 100, 150, and 200 μm were fabricated, forming a circular cross section, as shown in Fig. 2(b). The points shown on the circular cross section are the laser ablation points caused by pauses in the helical movement process.

## Waveguide laser operation

**Figure 3:**
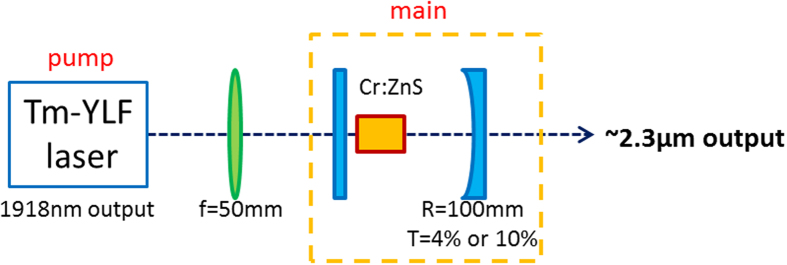
Schematic diagram of the Cr:ZnS waveguide laser.

The Cr:ZnS polycrystalline channel waveguide sample without an anti-reflective coating was built in a compact plane-concave cavity. Figure 3 presents the experimental setup of the Cr:ZnS polycrystalline waveguide laser. The optical pumping sources utilized a Tm:YLF laser (lasing at 1918 nm), which was pumped by a fiber-coupled diode laser (with a diameter of 400 μm and numerical aperture, NA = 0.22) at 793 nm. Water-cooled copper blocks were used to cool the crystals at a temperature of 16 °C, all of which were wrapped using indium foil. For the case of the Tm:YLF pump laser at 1918 nm, the maximum pump power and the emission bandwidth were approximately 3.68 W and 15 nm, respectively. The beam profiles of the Tm:YLF lasers were similar to the Gaussian TEM_00_ profile when near the maximum pump power.

Between the pump section and the main section, an f = 50 mm lens was adopted in order to focus the 1918 nm pump laser beam to a waist size of approximately 200 μm within the Cr:ZnS channel waveguides using helical writing. The resonator of the Cr:ZnS waveguide laser consisted of a plane dichroic input mirror (high transmission at 1800–2200 nm and high reflectivity (HR) at 2100–2800 nm) and a spherical dichroic output coupler with different transmittances (R = 100 mm, HR at 2100–2800 nm, and T = 4% and 10% at 2450 nm).

## Results and Discussions

**Figure 4:**
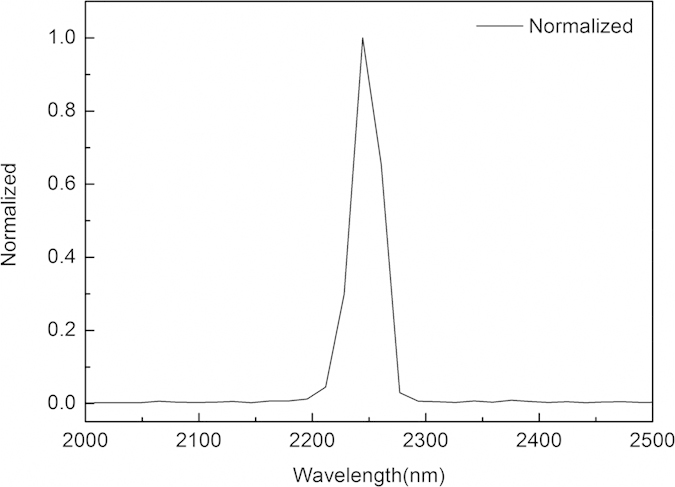
Normalized spectrum of Cr:ZnS waveguide laser pumping using a 1918 nm Tm:YLF laser.

**Figure 5:**
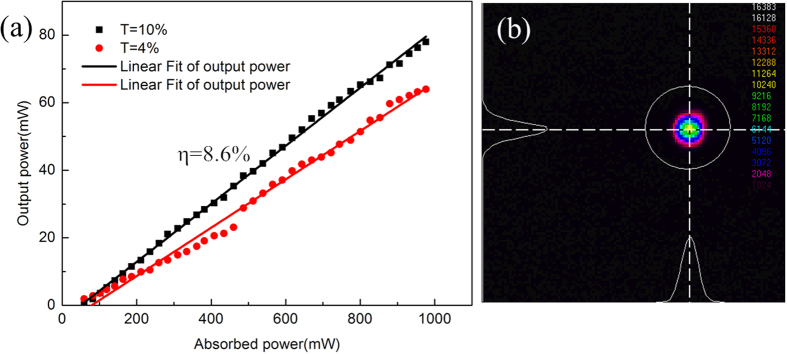
(**a**) Output power versus absorbed pump power using an output coupler with transmittances of T = 10% and T = 4%, lasing at 2244 nm. (**b**) Near-field beam profile of the Cr:ZnS waveguide laser, pumping at 1918 nm.

The laser output was observed for various structures, but the best performance was obtained for a waveguide with a diameter of 200 μm, with inscribed at a translation velocity of 1 mm·s^−1^. The spectrum was observed with a central wavelength at 2244 nm with a full width at half maximum (FWHM) of 30 nm, as shown in Fig. 4. The Cr:ZnS waveguide laser power output as a function of absorbed pump power is shown in Fig. 5 for the different output coupler transmittances (T = 4%, 10%).

From Fig. 5(a), we observe that the maximum power output and the optical-to-optical slope efficiency with respect to the absorbed pump power were 78 mW at 2244 nm and 8.6%, respectively, by 1918 nm Tm:YLF laser pumping and transmission of the output mirror of T = 10%. The threshold pump power was 29.17 mW. When using a transmission of the output mirror of T = 4%, the optical-to-optical slope efficiency with respect to the absorbed pump power was 7.1%. The maximum power output was 64 mW at 2244 nm, with a threshold pump power of 26 mW. The total inset loss was 7.43 dB including the propagation loss, Fresnel loss, and coupling loss. The input laser beam is focused approximate to the diameter of waveguide, so that the coupling loss C can be neglected. According to the following equations, the propagation loss of Cr:ZnS waveguide structure was approximately 0.38 dB/cm at 2.35 μm.


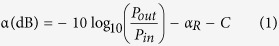



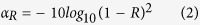



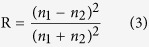


where α_R_ is the Fresnel loss, C is the coupling loss, P_out_ and P_in_ are the output and input power, R is the reflectivity, n_1_ and n_2_ are the refractive index of air (n_1_ = 1) and Cr:ZnS substrate (n_2_ = 2.27), respectively.

**Figure 6:**
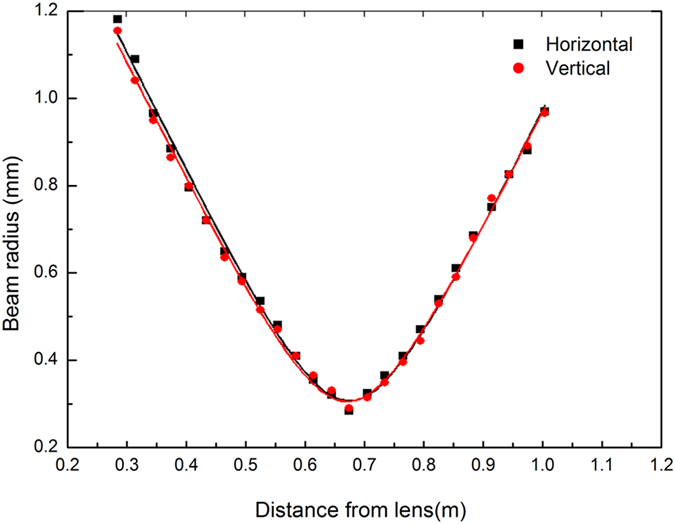
M^2^ beam quality for 1.1 W of pump power.

Nonetheless, the slope efficiency of laser output is inefficiency due to the surface of sample is without AR coating. The beam profiles for 1918 nm pumping are shown in Fig. 5(b), which were measured using an infrared-sensitive camera (Pyrocam III, Spirion). The beam profile of the Cr:ZnS waveguide laser was similar to the Gaussian TEM_00_ profile when near the maximum pump power. The horizontal polarization (p) of laser is 52.88% of the laser output energy, and the vertical polarization (s) is 47.12%. Figure 6 indicates the beam quality of the laser. The M_x_^2^ = 1.21 and M_y_^2^ = 1.19 values were measured and calculated along the axes orthogonal (x) and parallel (y) to the inscription, in the laser propagation direction. These results reveal that the channel waveguides we employed can efficiently guide and be used to improve laser beam quality.

## Summary

We have demonstrated the operation of a Cr:ZnS channel waveguide laser with a 8.6% slope efficiency and a 78 mW power output. Cr:ZnS waveguide laser emission at 2244 nm with a 30 nm FWHM line width was observed. Further optimization of the waveguide structures in order to reduce signal propagation losses and further modification in the form of Cr^2+^ doping in order to increase pump power absorption will be attempted in future work. This Cr:ZnS waveguide laser paves the way for the development of a compact, mid-infrared, tunable laser.

## Additional Information

**How to cite this article**: Peng, Y.-P. *et al.* Mid-infrared laser emission from Cr:ZnS channel waveguide fabricated by femtosecond laser helical writing. *Sci. Rep.* **5**, 18365; doi: 10.1038/srep18365 (2015).
